# Quality of Home Health Agencies Serving Traditional Medicare vs Medicare Advantage Beneficiaries

**DOI:** 10.1001/jamanetworkopen.2019.10622

**Published:** 2019-09-04

**Authors:** Margot L. Schwartz, Cyrus M. Kosar, Tracy M. Mroz, Amit Kumar, Momotazur Rahman

**Affiliations:** 1 School of Public Health, Health Services, Policy and Practice, Brown University School of Public Health, Providence, Rhode Island; 2 Rehabilitation Medicine, University of Washington, Seattle; 3 Physical Therapy, Northern Arizona University, Phoenix

## Abstract

**Importance:**

Medicare Advantage (MA) enrollment is increasing, with one-third of Medicare beneficiaries currently selecting MA. Despite this growth, it is difficult to assess the quality of the health care professionals and organizations that serve MA beneficiaries or to compare them with health care professionals and organizations serving traditional Medicare (TM) beneficiaries. Elderly individuals served by home health agencies (HHAs) may be particularly susceptible to the negative outcomes associated with low-quality care.

**Objective:**

To compare the quality of HHAs that serve TM and MA beneficiaries.

**Design, Setting, and Participants:**

This cross-sectional, admission-level analysis used data from 4 391 980 home health admissions identified using the Outcome and Assessment Information Set (most commonly known as OASIS) admission assessments of Medicare beneficiaries in 2015 from Medicare-certified HHAs. A multinomial logistic regression model was used to assess whether an association existed between the Medicare plan type and HHA quality. The model was adjusted for patient demographics, acuity, and characteristics of the zip codes. Sensitivity analyses controlled for zip code fixed effects. The present analysis was conducted between October 2018 and March 2019.

**Exposures:**

Home health users were classified as TM or MA beneficiaries using the Master Beneficiary Summary File. The MA beneficiaries were further classified as enrolled in a high- or low-quality MA plan on the basis of publicly reported MA star ratings.

**Main Outcomes and Measures:**

Quality of HHA derived from the publicly reported patient care star ratings: low quality (1.0-2.5 stars), average quality (3.0-3.5 stars), or high quality (≥4.0 stars).

**Results:**

Of 4 391 980 admissions, most (75.5%) were for TM beneficiaries (mean [SD] age, 76.1 [12.2] years), with 16.6% of beneficiaries enrolled in high-quality MA plans (mean [SD] age, 77.8 [10.0] years) and 7.9% in low-quality MA plans (mean [SD] age, 74.4 [11.4] years). Individuals enrolled in low-rated MA plans were most likely to be nonwhite (percentages of nonwhite individuals in TM, 14.3%; in high-quality MA, 19.8%; and in low-quality MA, 36.5%) and dual Medicare-Medicaid eligible (percentages for dual eligible in TM, 30.5%; in high-quality MA, 19.5%; and in low-quality MA, 43.3%). Among TM beneficiaries, 30.4% received care from high-quality HHAs, whereas 17.0% received care from low-quality HHAs. Compared with TM beneficiaries, those in a low-quality MA plan were 3.0 percentage points (95% CI, 2.6%-3.4%) more likely to be treated by a low-quality HHA and 4.9 percentage points (95% CI, −5.4% to −4.3%) less likely to be treated by a high-quality HHA. The MA beneficiaries in high-quality plans were also less likely to receive care from high-quality vs low-quality HHAs (−2.8% [95% CI, −3.1% to −2.2%] vs 1.0% [95% CI, 0.7%-1.3%]).

**Conclusions and Relevance:**

Compared with TM beneficiaries, MA beneficiaries residing in the same zip code enrolled in either high- or low-quality MA plans may receive treatment from lower-quality HHAs. Policy makers may consider incentivizing MA plans to include higher-quality HHAs in their networks and improving patient education regarding HHA quality.

## Key Points

**Question:**

Do Medicare Advantage beneficiaries receive a different quality of care from home health agencies than traditional Medicare beneficiaries do?

**Findings:**

In this cross-sectional study of more than 4 million home health agency admissions, Medicare Advantage beneficiaries were significantly less likely than traditional Medicare beneficiaries to receive treatment from high-quality home health agencies. Relative to traditional Medicare beneficiaries, the rate of receiving high-quality home health agency care was 4.9 percentage points lower for those enrolled in low-quality Medicare Advantage plans and 2.8 percentage points lower for those in high-quality Medicare Advantage plans.

**Meaning:**

Policy makers may consider incentivizing Medicare Advantage plans to include high-quality home health agencies in their networks and improving patient education regarding home health agency quality.

## Teaser

This cross-sectional study compares the quality of home health agencies that serve traditional Medicare beneficiaries vs beneficiaries enrolled in high-quality or low-quality Medicare Advantage plans.

## Introduction

The proportion of Medicare beneficiaries enrolled in Medicare Advantage (MA) increased from 13% in 2004 to 33% in 2017.^1^ Despite this growth, we know little about the quality of health care professionals serving MA beneficiaries compared with those serving traditional Medicare (TM) beneficiaries because of the incompleteness of MA claims data.

Both MA and TM beneficiaries use Medicare-certified home health (HH) services more than in the past. Between 2001 and 2015, Medicare spending for HH more than doubled to $18 billion.^2^ Of the 3.5 million^2^ Medicare beneficiaries who receive HH annually, half are older than 75,^3^ and all are homebound because of severe illness or functional limitation. The high risk of adverse outcomes in this vulnerable population^4^ may be exacerbated by receiving care from low-quality home health agencies (HHAs).

The inability to assess the quality of HHAs serving MA beneficiaries is concerning. Although MA plans are required to cover the same minimum health care services as TM, MA beneficiaries receive care from their plan’s network of preferred health care professionals and organizations, whereas TM beneficiaries may select any Medicare-certified health care professional and organization. Thus, similar to what has been observed in the private insurance market,^5,6,7^ MA plans may form networks with lower-quality HHAs that are willing to accept lower prices.

The objective of the present study is to compare the quality of HHAs serving MA and TM beneficiaries. We used HH assessment data to overcome the absence of MA claims and compared publicly reported quality ratings of HHAs serving MA and TM beneficiaries.

## Methods

### Study Design and Population

We conducted a cross-sectional, admission-level analysis of the association between Medicare type and HHA quality. We identified 5 071 922 Medicare HH admissions in 2015 by using Outcome and Assessment Information Set (OASIS) admission assessments. These assessments capture demographic and clinical data for all patients treated by Medicare- or Medicaid-certified HHAs. Admissions with missing data for any study variable were excluded, for a final sample of 4 391 980 admissions. There were more excluded admissions for MA (22.7%) than TM (9.8%) beneficiaries because of missing MA plan star ratings. In sensitivity analyses, thess omissions were not associated with our overall findings regarding differences in HHAs serving MA vs TM beneficiaries. Our analysis was conducted between October 2018 and March 2019. This study followed the Strengthening the Reporting of Observational Studies in Epidemiology (STROBE) reporting guideline for cross-sectional studies. This study was approved by the institutional review board of Brown University, which also waived the need to obtain informed consent under 45 CFR 46 because the research involved no more than minimal risk to human participants, did not adversely affect the rights and welfare of the participants, and could not practicably be conducted without the waiver.

### Outcome

We used publicly reported quality of care star ratings, published on the Centers for Medicare & Medicaid Services HH Compare website^8^ in July 2015 to represent HHA quality. The HHA star ratings summarize performance on 6 risk-adjusted outcome measures and 3 process-of-care measures.^9^ Ratings are reported in half-star intervals ranging from 1 star (lowest quality) to 5 stars. We classified HHAs into 3 quality categories: low (1.0-2.5 stars), average (3.0-3.5 stars), and high (≥4.0 stars).

### Explanatory Variable

We classified beneficiaries as MA or TM based on enrollment status recorded in the Master Beneficiary Summary File. We further classified MA beneficiaries according to the quality of their MA plans by merging MA plan identifications obtained from the Healthcare Effectiveness Data and Information Set with publicly reported MA star ratings. Beneficiaries were assigned to 1 of 3 categories: TM, low-quality MA plan (1.0-3.5 stars), or high-quality MA plan (≥4.0 stars).

### Covariates

Analyses were adjusted for patient demographics, baseline function, prior conditions, and zip code characteristics (Table 1) obtained from the Master Beneficiary Summary File, OASIS, and Centers for Medicare & Medicaid Services rural indicator file. To control for differential access to services, we determined the distance to the nearest low-, average-, and high-quality HHA by using ellipsoidal distances between the centroid of each patient’s zip code and the centroid of the zip code of each HHA.

**Table 1.** Characteristics of Medicare Home Health Patients by Plan Type

Characteristic	No. (%) of Patients		
	TM	MA	
		Low Quality	High Quality
Total No.	3 316 163	344 684	731 133
Patient characteristics			
Age, mean (SD), y	76.1 (12.2)	74.4 (11.4)	77.8 (10.0)
Female	2 037 932 (61.5)	216 671 (62.9)	454 451 (62.2)
Race/ethnicity			
Black	423 432 (12.8)	71 596 (20.8)	82 898 (11.3)
Other	348 598 (10.5)	54 051 (15.7)	61 999 (8.5)
Dual Medicare-Medicaid eligible	1 010 355 (30.5)	149 291 (43.3)	142 384 (19.5)
End-stage renal disease	125 276 (3.8)	9457 (2.7)	16 776 (2.3)
Home health use in year prior to admission	1 439 209 (43.4)	132 077 (38.3)	270 349 (37.0)
Inpatient discharge in prior 2 wk^a^			
Nursing facility	28 199 (0.9)	2621 (0.8)	36 603 (0.8)
SNF/transitional care unit	523 848 (15.8)	51 770 (15.0)	142 082 (19.4)
Acute care hospital	1 363 402 (41.1)	158 389 (46.0)	344 356 (47.1)
Long-term care hospital	21 033 (0.6)	2114 (0.6)	3654 (0.5)
Inpatient rehabilitation facility	232 282 (7.0)	24 594 (7.1)	42 026 (5.8)
Psychiatric facility	11 003 (0.3)	851 (0.3)	1503 (0.2)
Other	10 206 (0.3)	1107 (0.3)	3127 (0.4)
Prior conditions^a^			
Urinary incontinence	1 255 067 (37.9)	116 832 (33.9)	255 033 (34.9)
Catheter	67 380 (2.0)	6458 (1.9)	14 903 (2.0)
Intractable pain	489 173 (14.8)	46 891 (13.6)	99 805 (13.7)
Impaired decisions	633 500 (19.1)	55 201 (16.0)	118 155 (16.2)
Disruptive behavior	58 331 (1.8)	4600 (1.3)	9753 (1.3)
Memory loss	434 292 (13.1)	35 067 (10.2)	90 278 (12.4)
Ventilator use	3607 (0.1)	326 (0.1)	536 (0.1)
Functional scale score, mean (SD)^b^	3.3 (1.4)	3.1 (1.4)	3.1 (1.4)
Characteristic by patient zip code, mean (SD)			
Distance to nearest HHA by quality, miles^c^			
Low	40.2 (184.1)	39.4 (202.3)	31.0 (170.6)
Average	8.2 (32.7)	7.7 (33.9)	7.1 (25.2)
High	35.1 (159.7)	40.4 (175.3)	36.7 (172.7)
% Dual Medicare-Medicaid eligible in zip code	24.4 (12.0)	27.4 (12.8)	23.7 (11.2)
Zip code MA penetration	28.4 (1.3)	33.8 (12.4)	37.8 (13.3)
Rural county, %	18.1	15.4	12.6

Abbreviations: HHA, home health agency; MA, Medicare Advantage; OASIS, Outcome and Assessment Information Set; SNF, skilled nursing facility; TM, traditional Medicare.

^a^ Inpatient discharge and prior conditions are identified using check boxes in the OASIS assessments.

^b^ Represents performance across OASIS function items. Possible scores range from 0 to 8, with 0 representing complete independence and 8 indicating inability to a complete tasks such as ambulation, transferring, and grooming.

^c^ To convert miles to kilometers, multipy by 1.6.

### Statistical Analysis

We used multinomial logistic regression with zip code–clustered errors to assess the association between Medicare plan type and HHA quality. To facilitate interpretation, we present the marginal effect of MA plan type on HHA quality. This was interpreted as the absolute difference in the probability of receiving care from a low- or high-quality HHA if a patient enrolled in a low- or high-quality MA plan instead of TM, holding other characteristics constant.

We were unable to use zip code fixed effects in a multinomial model; therefore, we could not fully account for differences by neighborhood, which may be associated with patient selection and outcomes.^10,11^ We conducted sensitivity analyses using separate linear regression models (one with the outcome high-quality HHA and one with the outcome low-quality HHA) and controlled for zip code fixed effects and patient characteristics. Analyses were conducted using Stata, version 14 (StataCorp LLC).

## Results

Table 1 gives the characteristics of the study population stratified by Medicare plan type. The majority (75.5%) of admissions were for TM beneficiaries (mean [SD] age, 76.1 [12.2] years), whereas 16.6% of beneficiaries were enrolled in the high-quality MA plan (mean [SD] age, 77.8 [10.0] years) and 7.9% in the low-quality MA plans (mean [SD] age, 74.4 [11.4] years). Individuals enrolled in low-rated MA plans were most likely to be nonwhite (14.3% of TM beneficiaries, 19.8% in high-quality MA plans, and 36.5% in low-quality MA plans) and dual Medicare-Medicaid eligible (30.5% of TM beneficiaries, 19.5% in high-quality MA plans, and 43.3% in low-quality MA plans).

The Figure uses polynomial regression curves to display the rate of high-quality HHA use in association with individuals’ distances to the nearest high-quality HHA across Medicare plan types. The TM beneficiaries were more likely than both types of MA beneficiaries from the same neighborhood to receive care from a high-quality HHA. Differences across plan types decreased as distance to the nearest high-quality HHA increased, suggesting that if a high-quality HHA is too far, all residents are unlikely to receive high-quality HHA care.

**Figure.**
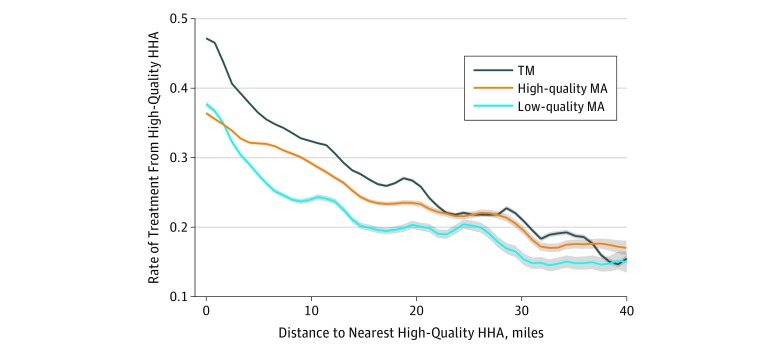
Rate of Treatment From a High-Quality Home Health Agency (HHA) by Distance to Nearest High-Quality Agency Across Medicare Plan Types High-quality HHAs receive 4 to 5 stars. Shading indicates 95% CIs. To convert miles to kilometers, multipy by 1.6. MA indicates Medicare Advantage; TM, traditional Medicare.

Among TM beneficiaries, the proportion receiving care from low-quality HHAs was 17.0%, and the proportion receiving care from high-quality HHAs was 30.4% (Table 2). After adjustment using multinomial logistic regression, compared with TM beneficiaries, those in a low-quality MA plan were 3.0 percentage points (95% CI, 2.6%-3.4%) more likely to be treated by a low-quality HHA and 4.9 percentage points (95% CI, −5.4% to −4.3%) less likely to be treated by a high-quality HHA. The MA beneficiaries in high-quality plans were also less likely to receive care from high-quality HHAs, although the magnitude of the difference from TM was smaller (adjusted difference in likelihood of treatment from low-quality HHA, 1.0% [95% CI, 0.7%-1.3% vs adjusted difference in treatment from high-quality HHA, −2.8% [95% CI, −3.1% to −2.2%]).

**Table 2.** Association Between Medicare Plan Type and Changes in Rate of Treatment by Low- and High-Quality HHAs

Quality of HHA	TM, Distribution, %	Low-Quality MA Plan				High-Quality MA Plan			
		Distribution, %	Unadjusted Difference From TM, %	Adjusted Difference, % (95% CI)		Distribution, %	Unadjusted Difference From TM, %	Adjusted Difference, % (95% CI)	
				Multinomial Model^a^	Linear Model^b^			Multinomial Model^a^	Linear Model^b^
Low, <3 stars	17.0	23.5	6.50	3.0 (2.6 to 3.4)	2.3 (2.2 to 2.4)	18.3	1.30	1.0 (0.7 to 1.3)	2.2 (2.1 to 2.3)
High, 4-5 stars	30.4	22.6	−7.80	−4.9 (−5.4 to −4.3)	−2.0 (−2.2 to −1.9)	27.0	−3.40	−2.8 (−3.1 to −2.2)	−3.1 (−3.2 to −3.0)

Abbreviations: HHAs, home health agencies; MA, Medicare Advantage; TM, traditional Medicare.

^a^ Represents the marginal effects of our multinomial regression model assessing the association between Medicare plan type and quality of treating HHA. The model was adjusted for all variables listed in Table 1, and confidence intervals were clustered on zip code.

^b^ Represents estimates of the linear regression models (one with the outcome high-quality HHA and one with the outcome low-quality HHA). The linear models were adjusted for all patient characteristics listed in Table 1 as well as for zip code fixed effects.

After adjustment using linear regression models with zip code fixed effects, MA enrollees overall remained less likely than TM enrollees to receive care from high-quality HHAs. However, differences within MA enrollees by plan type were either eliminated or inverted. Compared with TM, the low-quality MA adjusted difference in likelihood of treatment from a low-quality HHA was 2.3% (95% CI, 2.2%-2.4%), and the adjusted difference in treatment from a high-quality HHA was −2.0% (95% CI, −2.2% to −1.9%). Compared with TM, the high-quality MA adjusted difference in likelihood of treatment from a low-quality HHA was 2.2% (95% CI, 2.1%-2.3%), and the adjusted difference in treatment from a high-quality HHA was −3.1% (95% CI, −3.2% to −3.0).

## Discussion

We found that MA enrollees were less likely to receive care from high-quality HHAs compared with their TM counterparts. We did not find that individuals enrolled in low-rated MA plans, who were more likely to be nonwhite and enrolled in Medicaid, received lower-quality HH care compared with individuals enrolled in high-quality MA plans after controlling for zip code. However, lower HHA quality for MA enrollees overall raises concerns about potential disparities in access to high-quality HH care and is consistent with prior research documenting greater use of low-quality skilled nursing facilities in the MA program.^12^

Lower HH quality for MA beneficiaries may be attributable to plans’ narrow HHA networks, which may include low-quality HHAs that are willing to accept lower prices. In fact, HHAs are excluded from MA network adequacy criteria.^13^ Revisions to these criteria may incentivize MA plans to expand networks and increase access to higher-quality HHAs. In addition, patient education and reforms in the acute discharge planning process may improve access to and information about postacute care quality for HH.^14,15,16^

Whether or not lower-quality HH or other postacute care translates to worse outcomes for MA enrollees is uncertain. The MA plans have strong incentives to coordinate care for enrollees because they receive capitated per beneficiary payments from Medicare. However, if MA enrollees are healthier or receive better care elsewhere, then MA plans may invest less in HH care. In addition, findings across studies examining TM and MA differences in patients’ posthospital outcomes are inconsistent and have not focused on patients receiving HH care.^17,18,19^

### Limitations

The present study had several limitations. We did not control for MA plan structure (eg, health maintenance organization vs preferred provider organization). In addition, we could not observe the HHA selection process and thus could not determine the primary mechanisms behind differences in HHA quality by plan type. Star ratings may not adequately capture quality of care, and the reliability and validity of these measures have been questioned.^20^ For example, a study showed that the rank ordering of facilities is sensitive to changes in risk adjustment methods.^21^ Although a recent rigorously designed study found that nursing homes with higher star ratings provide better quality care,^22^ future research that assesses the adequacy of home health star ratings is needed. Despite these limitations, our findings represent novel evidence regarding HHA quality by Medicare plan type during a time of substantial growth in both the MA and HH populations.

## Conclusions

The present study results indicated that compared with their TM counterparts, MA beneficiaries received treatment from lower-quality HHAs. Policy makers may consider incentivizing MA plans to include higher-quality HHAs in their preferred HHA networks and improving patient education regarding HHA quality.
